# Predicting potential SARS-CoV-2 mutations of concern via full quantum mechanical modelling

**DOI:** 10.1098/rsif.2023.0614

**Published:** 2024-02-07

**Authors:** Marco Zaccaria, Luigi Genovese, Brigitte E. Lawhorn, William Dawson, Andrew S. Joyal, Jingqing Hu, Patrick Autissier, Takahito Nakajima, Welkin E. Johnson, Ismael Fofana, Michael Farzan, Babak Momeni

**Affiliations:** ^1^ Department of Biology, Boston College, Chestnut Hill, MA, USA; ^2^ Université Grenoble Alpes, CEA, INAC-MEM, L Sim, Grenoble, France; ^3^ RIKEN Center for Computational Science, Kobe, Japan; ^4^ Department of Pediatrics, Harvard Medical School, Boston, MA, USA; ^5^ Center for Integrated Solutions for Infectious Diseases, The Broad Institute of MIT and Harvard, Cambridge, MA, USA; ^6^ Division of Infectious Disease, Boston Children's Hospital, Boston, MA, USA

**Keywords:** quantum mechanical modelling, *ab initio* modelling, SARS-CoV-2, spike protein, evolution, protein–protein interactions

## Abstract

*Ab initio* quantum mechanical models can characterize and predict intermolecular binding, but only recently have models including more than a few hundred atoms gained traction. Here, we simulate the electronic structure for approximately 13 000 atoms to predict and characterize binding of SARS-CoV-2 spike variants to the human ACE2 (hACE2) receptor using the quantum mechanics complexity reduction (QM-CR) approach. We compare four spike variants in our analysis: Wuhan, Omicron, and two Omicron-based variants. To assess binding, we mechanistically characterize the energetic contribution of each amino acid involved, and predict the effect of select single amino acid mutations. We validate our computational predictions experimentally by comparing the efficacy of spike variants binding to cells expressing hACE2. At the time we performed our simulations (December 2021), the mutation A484K which our model predicted to be highly beneficial to ACE2 binding had not been identified in epidemiological surveys; only recently (August 2023) has it appeared in variant BA.2.86. We argue that our computational model, QM-CR, can identify mutations critical for intermolecular interactions and inform the engineering of high-specificity interactors.

## 1. Introduction

Many viruses initiate entry into host cells through interactions with a receptor on the target cell's surface. Mutations may occur in the viral genome that enhance this interaction. If overall more fit, variants with such mutations can become dominant among the circulating strands. Over the course of the SARS-CoV-2 pandemic, several SARS-CoV-2 strains emerged that had modifications at the receptor binding domain (RBD) of the viral spike protein (S protein) [1–4]. The spike protein binds the human angiotensin converting enzyme 2 (hACE2) receptor to initiate SARS-CoV-2 infection. We aim to mechanistically understand how the spike protein interacts with hACE2 to predict which viral variants best exploit the chemical space, and how. Our analysis in this regard focuses on the SARS-CoV-2 primary receptor ACE2 and does not include known co-receptors such as NRP-1, AXL, HSPG, KREMEN1 and CD147 [5]*.* Under the assumption of increased spike–hACE2 binding strength as one of the drivers of viral adaptation to human hosts, we can identify which mutations are likely to characterize a future prominent SARS-CoV-2 variant. Developing such predictive mechanistic models can facilitate pre-emptive engineering of pharmacological countermeasures to block viral evolutionary trajectories towards stronger binding. Prepared and guided by this insight, vaccines or treatments can nip dangerous lineages in the bud, before they become dominant.

Predictive models based on quantum mechanics (QM), calculations can characterize intermolecular interactions at high resolution without requiring prior knowledge and parametrizations [6–10]. A full quantum mechanical environment can be simplified and made accessible by mapping the contributions to the amino acid level and characterization of the share of each residue to the overall binding energy between two proteins. Here, we employ the spike–hACE2 binding as a case study to illustrate that *ab initio* full QM models can predict intermolecular binding when thousands of atoms are involved.

We implement the full QM model using the QM complexity reduction (QM-CR) approach formalized in a recent work [11]. Starting from a representative 3D model of the molecules as input [12], the BigDFT program [13] calculates the electronic density matrix based on density functional theory to extract inter- and intra-molecular interactions. The strength of inter-residue interactions is quantified through the fragment bond order (FBO) [14]. FBO is calculated using the electronic structure of the system, in the vicinity of a given residue. FBO identifies the residues involved in a chemical interaction, namely the amino acids of the counter-ligand that share a non-negligible bond—above a set threshold—with the ligand. In contrast to a purely geometrical indicator, like the residue–residue distance, FBO allows agnostic identification of residues that contribute to the intermolecular interaction. Once a chemical interaction is identified, we assign to each residue a specific value in the overall contribution to the intermolecular binding. These quantities are outputs of the BigDFT code and represent two terms: (i) short range: attractive via chemical bond, which is non-zero only if the cross-fragment electronic clouds overlap; (ii) long range: electrostatic attractive/repulsive, defined from the electron distributions of each fragment; long-range terms allow identification of off-interface relevant residues. Ultimately, QM-CR provides a mechanistic *ab initio* representation of a given ligand–counter ligand interaction as the final output. A contact map summarizing relevant interactions between the Wuhan spike RBD and hACE2 is available in our previous work on QM-CR [11]. We perform experimental verification of the model's predictions via a fluocytometric assay of spike RBD–hACE2 binding.

## 2. Material and methods

### 2.1. Plasmid

The pCAGGS plasmid expressing SARS-CoV-2 Wuhan strain spike protein has previously been described [15]. Synthetic DNA of spike genes corresponding to variants of SARS-CoV-2 Omicron strains were obtained from Integrated DNA Technologies (IDT, Coralville, IA, USA). The variants (sequences available in electronic supplementary material, table S1) were cloned into pCAGGS to replace the SARS-CoV-2 Wuhan strain spike protein via KpnI/SacI digestion and T4 DNA ligation. The cloning strategy results in RBD fusion with human IgG1-Fc to generate recombinant RBD-Fc variants. HEK293T (human embryonic kidney; ATCC, Virginia, USA) were maintained in growth media DMEM-10x composed of Dulbecco's modified Eagle's medium (DMEM, Thermo-Fisher, MA, USA) supplemented with 2 mM glutamine (Thermo-Fisher), 1% non-essential amino acids (Thermo-Fisher), 100 U ml^−1^ penicillin, 100 µg ml^−1^ streptomycin (Thermo-Fisher), and 10% FBS (Thermo-Fisher) at 37°C in 5% CO_2_. HEK293T cell lines expressing hACE2 were previously described [15]. Parental cells were transduced with generated MLV virus, and cell lines that stably expressed hACE2 were selected and maintained with medium containing 3 µg ml^−1^ puromycin (Sigma, MI, USA). hACE2 expression was confirmed by immunofluorescence staining using mouse monoclonal antibody against c-Myc antibody 9E10 (Thermo-Fisher) and goat-anti-mouse IgG APC (Jackson ImmunoResearch Laboratories, Pennsylvania, USA).

### 2.2. Protein production and purification

Fc fusion protein production and purification has been previously described for RBD-Fc [16,17]. Briefly, HEK293T cells were cultured in DMEM-10x media. HEK293T cells (12 × 10^6^) were seeded in T175 flasks (Thermo-Fisher) and incubated overnight (37°C, 5% CO_2_) on the day before transfection. On the day of transfection, plasmid DNA was transfected using 70 µl of Transfectin Lipid Reagent (BioRad, CA, USA). Four or five T175 plates were prepared for each plasmid DNA. Twenty-four hours after initiation of transfection, culture media was replaced with serum free media (SFM) composed of DMEM-10x. Five days after transfection, culture supernatants were harvested and cells were pelleted by centrifugation (1500 rpm, 5 min). The resulting supernatant was incubated overnight with protein A agarose beads at 4°C with rotation. RBD-Fc was then purified by gravity flow using Econo-Glass column (BioRad). RBD-Fc elution was performed using 0.1 M glycine (pH 2.2) and the eluate was neutralized with 1.5 M Tris-HCl pH 8.8. Purified protein was dialysed overnight against pH 7.4 phosphate-buffered saline (PBS) using 10 000 molecular weight cut-off (MWCO) dialysis cassettes (BioRad). Finally, purified RBD-Fc proteins were concentrated using 10 000 MWCO Amicon centrifugation columns (BioRad) and stored at 4°C before use. Protein concentration was determined by spectrometry using a NanoDrop microvolume spectrometer (Thermo-Fisher).

### 2.3. Flow cytometry to test the binding of coronavirus RBD-Fc proteins to hACE2 receptor

HEK293T cell lines expressing hACE2 were cultured as previously described, in the presence of 3 µg ml^−1^ puromycin to select for hACE2 expression [15]. hACE2 expression was detected using mouse monoclonal antibody against c-Myc-FITC antibody (clone 9E10) from Thermo-Fisher. Original HEK293T cell lines were used as a control. Binding affinity of RBD-Fc [11] variants were detected by staining HEK293T-hACE2 cells as previously described [15] (electronic supplementary material, figure S3). Briefly, 10^6^ cells were incubated (15 min, room temperature) with 0.5 µg ml^−1^ of RBD-Fc variants in PBS containing 0.5% bovine serum albumin (BSA) (Thermo-Fisher). The cells were washed with PBS, 0.5% BSA and RBD-Fc binding was detected with goat anti-human-Ig-APC (Jackson ImmunoResearch Labs, Pennsylvania, USA). Data were acquired using flow cytometry using an LSRII (BD Biosciences, California, USA) and analysed using the FlowJo software (FlowJo, LLC).

### 2.4. Quantum mechanical calculations

We performed Kohn–Sham density functional theory [18] calculations using the BigDFT program with Hartwigsen–Goedecker–Hutter pseudo-potentials [19], a grid spacing of 0.4 atomic units, and the PBE exchange-correlation functional. Dispersion interactions were added using the D3 correction term [20]. In BigDFT, the Kohn–Sham orbitals are expanded in a set of *in situ* optimized, localized orbitals which are in turn represented by Daubechies wavelets [21]. Calculations were performed in gas phase, which was shown to perform adequately compared to the inclusion of implicit solvent for this system in our previous work [11], at finite electronic temperature using the CheSS library [22]. We post-process these QM calculations to compute interaction measures among the various components of the systems. Using operator representation, we define a general quasi-observable $O_{FG}$ associated with two system fragments *F* and *G* as

$$O_{FG}=\mathrm{tr}(W_{F}OW_{G}K),$$

where *K* is the density matrix of the full system, *W* is the projection onto a given fragment, *O* is the operator of interest, and tr() is the trace of an operator. In this formulation, the FBO is defined as:

$$B_{FG}=\mathrm{tr}(W_{F}KW_{G}K).$$

The short-range (chemical/contact) interaction is defined as the sum of the contact component of all interactions between fragments *F* from one molecule and fragments *G* from the other molecule (*H* is the Kohn–Sham Hamiltonian):

$$E_{FG}^{ct}=\mathrm{tr}(W_{F}HW_{G}K),$$

with the empirical D3 correction mentioned above. Such a term correlates with the FBO and provides an indication of the chemical coupling between fragment *F* and fragment *G*.

The long-range interaction term is calculated from the electrostatic interaction between the charge densities (*ρ*) of *F* and *G*:

$$E_{FG}^{el}=\;\int drdr^{\prime }\;\frac{\rho_{F}(r)\rho_{G}(r^{\prime })}{\left|r-r^{\prime }\right|},$$

which is approximated as the sum of atom-centred multipoles (up to second order) computed from the electronic and ionic densities. When extended to the systems' constituents, such a long-range term constitutes the first-order approximation of the three-point interaction (binding) energy, as previously demonstrated [23].

## 3. Results

Throughout this work, we use the 6M0J entry in the RCSB database [12] as our starting crystallographic structures. We assign pH 7 to histidine protonation and other titratable residues via the PDBFixer tool in OpenMM [24]. To generate the crystal structures with point mutations, we optimize the geometry through structure relaxations via the AMBER FF14SB force field [25], also available in the OpenMM package. This procedure aims to estimate to the first-order how binding changes when a number of mutations are introduced relative to the original structure (a unique set of mutations defines a variant). While this structure relaxation does not cover all possible conformations, we expect it to offer a reasonable approximation, as it has been successfully used for the spike–hACE2 binding in a previous work [11].

### 3.1. Compared to Wuhan spike RBD, vOmicron binds to hACE2 more strongly

**Figure 1.**
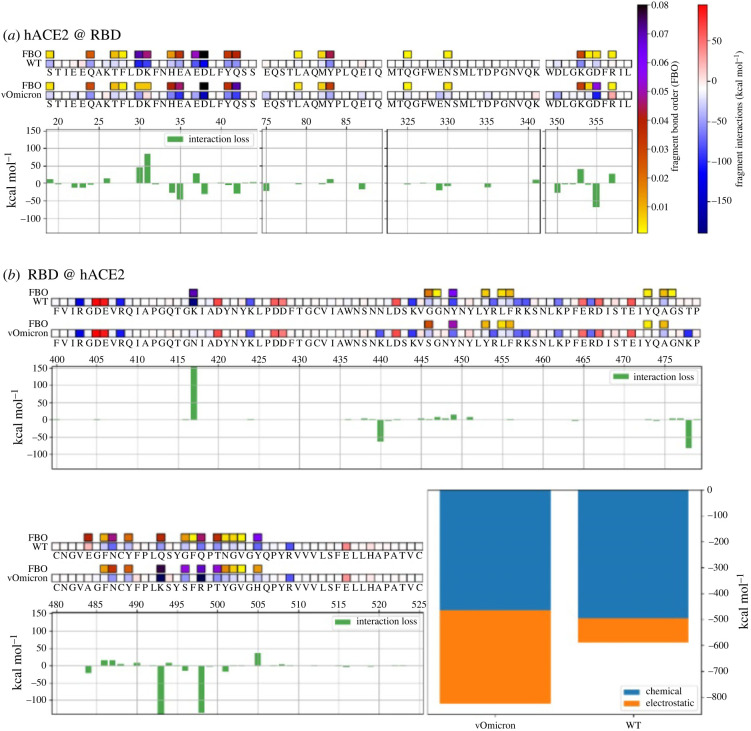
Mechanistic characterization of spike-hACE2 binding for the Wuhan (WT) and the Omicron variant spikes. Data are plotted on hACE2 (*a*) and the different viral spikes (*b*). Namely, we consider the Wuhan (WT) and Omicron spikes. Amino acids are represented by the corresponding letters and numbered on the histogram's horizontal axis. Their overall effect on the ligand is indicated by red/repulsive and blue/attractive squares; the energy scale is identical throughout all figures. Interface residues are highlighted by bars on top of the interaction values, and darker colours indicate stronger interface values. Histograms underneath the sequences show the relative change in binding energy. Bottom right histograms represent the overall binding energy of hACE2 with the Wuhan and Omicron variants, partitioned into chemical or electrostatic contributions.

We imposed the following mutations to represent the Omicron variant's spike RBD: K417N, N440K, G446S, S477N, T478K, E484A, Q493K, G496S, Q498R, N501Y and Y505H. We opted to leave out G339D, S371L, S373P, and S375F, since they are far from the interface and not electrostatically active. The resulting strain is based on the mutations found in the Omicron variant, but is simplified because it lacks some of the mutations that are unlikely to affect hACE2 binding. We call this strain virtual Omicron (vOmicron, or vOm for short) and will use it both for our computational investigations and for experimental validations. QM-CR highlights how the mutations present in the vOmicron variant lead to a different interaction pattern with hACE2 compared to the original strain. We characterized the role of each mutation in the interaction rearrangement according to the hotspot regions they define and depending on their stabilizing power on and off the interface. vOmicron is predicted to have a much stronger binding enthalpy than Wuhan (figure 1, lower right panel). The interaction pattern of vOmicron with hACE2 (figure 1*a,b*) highlights a comparable chemical/short-range contribution to binding, but a higher long-range/electrostatic contribution which strongly favors vOmicron over Wuhan, overall.

### 3.2. Some spike RBD mutations can further strengthen its binding to hACE2

**Figure 2.**
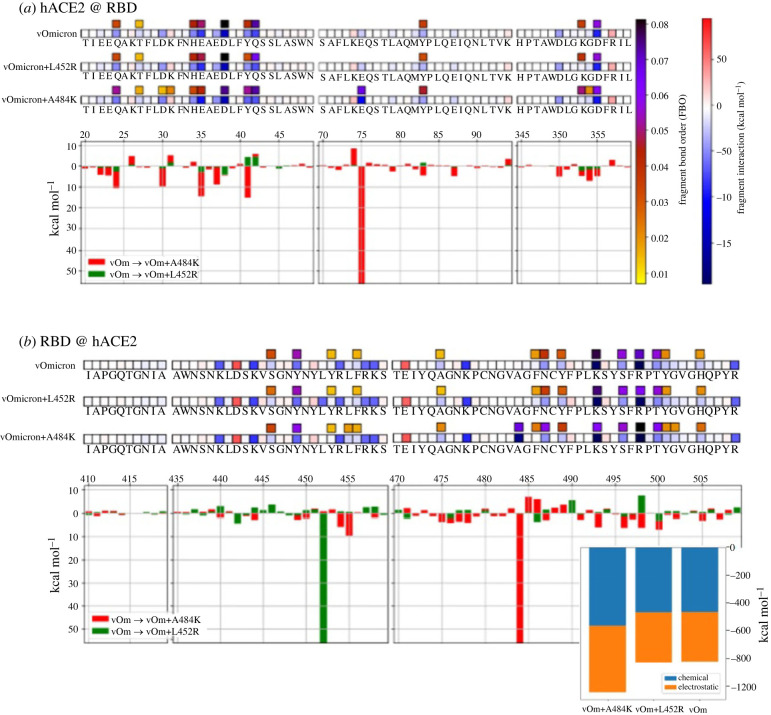
Characterization of the virtual mutation L452R and A484K on the vOmicron variant. Data are plotted on the hACE2 primary structure (*a*), and on the Wuhan spike RBD (*b*), when binding to the vOmicron and the putative vOmicron + L452R or vOmicron + A484K variants. Amino acid residues are labelled with letters and numbered. Interface residues are highlighted with a yellow bar; red and blue tiles are repulsive and attractive residues, respectively.

We tested whether QM-CR can predict the strength of hACE2 binding to hypothetical variants that may arise in the future. To this end, we examined two mutants that, at the start of our effort [26], had not yet emerged in epidemiological surveys: (a) vOmicron + L452R, incorporating a mutation from the formerly dominant Delta variant (this mutation later emerged in Omicron BA.4/5); and (b) vOmicron + A484K (emerged in August 2023 in BA.2.86), incorporating a mutation from the once common Beta and Gamma variants and known to enhance hACE2 binding and antibody evasion [27]. QM-CR simulations predict that L452R does not substantially alter the spike–hACE2 binding strength; the increase in the long-range contribution is cancelled out by energy losses along the spike structure. Conversely, QM-CR predicts that A484K dramatically increases the electrostatic component of the S-hACE2 binding, without compromising pre-existing hotspots (figure 2). In addition to the differences among the tested variants in overall binding energy, the interaction pattern of the vOmicron + A484K includes new residues participating in the interaction, such as L455, K484 and G502 on the RBD side.

### 3.3. Experiments validate the QM-CR predicted strength of spike RBD binding to hACE2

**Figure 3.**
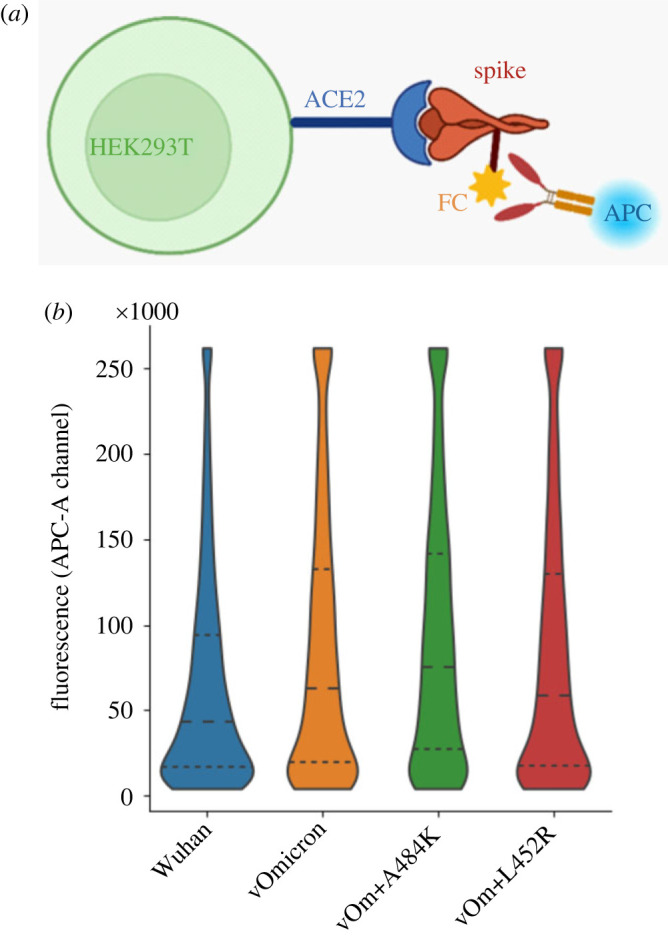
Spike–hACE2 Binding assays. For the binding experiment, the spike protein's FC tag is bound by an antibody carrying the APC fluorescent label (*a*). The four different spike protein binding performances are expressed in violin plots (*b*), with the dashed line being the median fluorescence intensity after binding. The dotted lines show the upper and lower quartiles of the measured fluorescence values.

**Figure 4.**
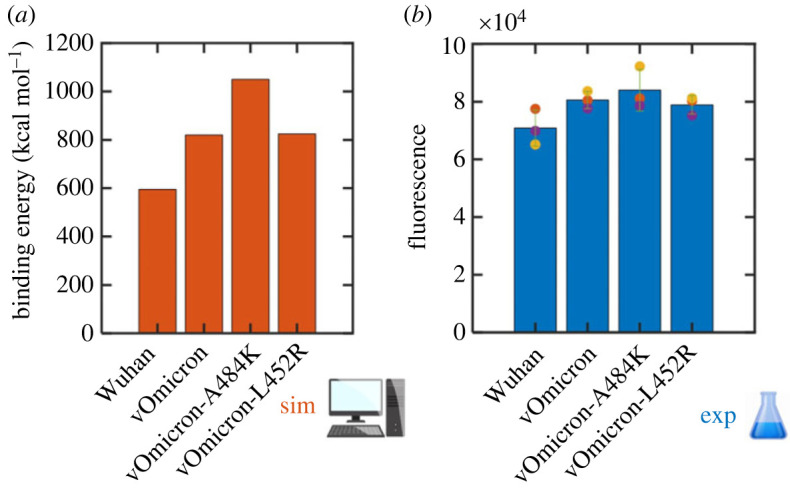
Computational predictions are consistent with the results of binding experiments. Energy of the four simulated spike structures' binding to the hACE2 as predicted by QM-CR (*a*). Fluorescence results are plotted (*b*) as the average of MFI across three biological replicates. Data from each replicate are presented by dots of the same colour.

To experimentally validate QM-CR predictions, we generated the RBD S-Fc fusion expression constructs of the Wuhan, vOmicron, vOmicron + A484K and vOmicron + L452R spike proteins. The constructs were cloned into a plasmid expressing codon-optimized Wuhan spike protein (pCAGGS) [28] to produce spike proteins of different variants. We then tested the affinity of these variants by assessing their binding to HEK293T cells that express hACE2 on their surface. Using flow cytometry, we compared the strength of binding by assessing the mean fluorescence intensity (MFI) of cells bound to fluorescently stained spike RBD (figure 3 and electronic supplementary material, figures S1–S2). We confirmed that the median fluorescence intensity exhibits the same trend (electronic supplementary material, figure S4). In the experiment, vOmicron + A484K displays the highest number of binding events, which correlates with QM-CR predictions (figure 4). vOmicron + L452R, vOmicron and Wuhan all follow the predicted trend (figure 4). A Kruskal–Wallis test of the experimental APC-A readouts for the four groups in figure 4 rejects the null hypothesis that the distributions are similar (*p* = 6 × 10^−147^). All pairwise comparisons of flow cytometry data for different spike variants show statistically significant differences (Mann–Whitney *U*-test). Details of the *p*-values for pairwise flow cytometry data comparisons are in the supplementary information (electronic supplementary material, table S2). Fluorescence background values are minimal (available in the electronic supplementary material); overall, the relevant quarter of the distribution is populated with less than 0.2% data points in the absence of the secondary antibody.

## 4. Discussion

In this work, we performed a full QM simulation of intermolecular binding to draw mechanistic insight about the role of each amino acid. We also predicted the effect of amino acid mutations on binding at and away from the mutated residue. Our work expands on the existing work [6–11] on the binding of SARS-CoV-2 spike protein to the human ACE2 receptor. We incorporated mutations relevant to Omicron variants, without making *a priori* assumptions about interacting residues (thus, we included approx. 13 000 atoms in our simulations). We show that, in the context of the SARS-CoV-2 spike of the Omicron variant and its mutated versions, QM-CR predictions of the binding strength match experimental validations. We should clarify that the QM-CR approach involves a post-processing method, intended to offer a biologically useful interpretation of the QM data. The underlying QM calculations remain unbiased and unconstrained: mapping the effect on amino acids (or, more generally, molecular fragments) does not introduce any additional approximation.

Our modelling and analysis are focused on the binding effect between the spike and hACE2 as its primary receptor. Such an analysis does not predict the effects a given mutation may have on binding to other host receptors [5] or on other stages of viral infection and replication, especially when conformational changes are implied. As such, we do not expect our predictions on binding strength to fully match the infectivity of specific SARS-CoV-2 variants. This is well exemplified by the case of mutation L452R which is known to improve infectivity via increased fusogenicity [29], a mandatory event for cell entry after binding. In line with previous research, our simulations do not predict a binding advantage for L452R.

QM-CR relies on a crystal structure as its input, and is therefore dependent on the availability of the crystal structure at an adequate resolution. Such crystal structures ideally include the bound substrate of interest; however, docking simulations can be performed to infer a ligand's position to inform the model. In our assessment of mutants, we have further assumed that imposing these mutations does not considerably affect the structure; therefore, we have approximated the mutated structure by performing a local optimization around the existing structure [11]. If there are major changes in the shape of a protein as a consequence of mutations, a more representative crystal structure of such variants is needed as the starting point.

## 5. Conclusion

In its present stage, QM-CR is a powerful tool if focused on binding energy/affinity. It is therefore of potential use in identifying candidates for molecular inhibitors, antibodies, or any other interactors expected to bind and stick to a receptor/substrate. We believe QM-CR can be of particular importance in the context of drug discovery and immunology, especially to screen for promising candidate molecules or to refine existing candidates. With minimal requirements for prior knowledge about interacting molecules, QM-CR can be applied as a general approach to a wide range of biological processes when a crystal structure (or a reliable estimate of it) is available. In the case of SARS-CoV-2, QM-CR can inform the design of specific molecular inhibitors against hypothetical variants that may emerge—an important aspect of pandemic readiness. Case in point is the recent emergence of SARS-CoV-2 variant BA.2.86 with the A484K mutation in August 2023 epidemiological surveys—a mutation that stood out as a strong candidate in our simulation performed in December 2021. We argue that its recent emergence supports the predictive power of QM-CR.

## Data Availability

The flow cytometry raw data from biological replicates and corresponding analysis codes are available from the Zenodo repository: https://doi.org/10.5281/zenodo.10459824 [30]. Additional data are provided in the electronic supplementary material [31].
